# Individual differences in intracortical inhibition predict action control when facing emotional stimuli

**DOI:** 10.3389/fpsyg.2024.1391723

**Published:** 2024-06-12

**Authors:** Thomas Quettier, Giuseppe Ippolito, Lorenzo Però, Pasquale Cardellicchio, Simone Battaglia, Sara Borgomaneri

**Affiliations:** ^1^Center for Studies and Research in Cognitive Neuroscience, Department of Psychology “Renzo Canestrari”, Cesena Campus, Alma Mater Studiorum Università di Bologna, Cesena, Italy; ^2^Laboratory of Cognitive Neuroscience, Department of Languages and Literatures, Communication, Education and Society, University of Udine, Udine, Italy; ^3^Physical Medicine and Rehabilitation Unit, IRCCS Istituto Giannina Gaslini, Genoa, Italy; ^4^Department of Psychology, University of Torino, Torino, Italy

**Keywords:** action control, stop signal task, fearful body postures, transcranial magnetic stimulation, intracortical inhibition and facilitation

## Abstract

Efficient inhibitory control in the context of prepotent actions is vital. However, such action inhibition may be profoundly influenced by affective states. Interestingly, research indicates that action control can be either impaired or improved by emotional stimuli. Thus, a great deal of confusion surrounds our knowledge of the complex dynamics subtending emotions and action control. Here, we aimed to investigate whether negative stimuli, even when non-consciously presented and task-irrelevant, can affect action control relative to neutral stimuli. Additionally, we tested whether individual differences in intracortical excitability may predict action control capabilities. To address these issues, we asked participants to complete a modified version of the Stop Signal Task (SST) in which fearful or neutral stimuli were subliminally presented before the go signals as primes. Moreover, we assessed participants’ resting-state corticospinal excitability, short intracortical inhibition (SICI), and intracortical facilitation (ICF). Results demonstrated better action control capabilities when fearful stimuli were subliminally presented and interindividual SICI predicted stronger action inhibition capabilities. Taken together, these results shed new light on the intricate dynamics between action, consciousness, and motor control, suggesting that intracortical measures can be used as potential biomarkers of reduced motor inhibition in research and clinical settings.

## Introduction

Cognitive neuroscience has long been fascinated by the intricate ways in which emotions influence complex cognitive functions, including action inhibition. From an operational point of view, the examination of action inhibition typically involves the utilization of the stop-signal task (SST), which is specifically devised to offer a sensitive assessment of the duration it takes for the brain to inhibit or suppress inappropriate motor responses (Logan, 1994; Verbruggen et al., 2019). In an SST, participants are requested to respond to a “go” stimulus. Nevertheless, on certain occasions, the go stimulus is succeeded by a “stop” signal, necessitating participants to refrain from continuing the ongoing action. In assessing the participant’s performance on the SST, the stop signal reaction time (SSRT), an indicator of inhibition, is calculated according to the concept proposed by Logan et al. (1984). Several studies using SST have aimed to uncover the influence of emotional stimuli on action control capabilities. However, findings are inconsistent, as these investigations collectively reveal that emotion can either impair, facilitate, or have no effect on action control (for a review see Battaglia et al., 2021). A factor that might have influenced the divergent outcomes is the varied functions of the emotional stimulus, such as being presented as a stop signal, go signal, or as a prime before the go signal. Additionally, the significance of the emotional stimulus for the SST plays a role. Emotional stimuli can be either task-relevant, necessitating explicit discrimination of emotional stimuli (e.g., Ding et al., 2021) or task-irrelevant (i.e., not requiring emotion discrimination; e.g., Sagaspe et al., 2011). Experiments employing SSTs that required emotion discrimination found that emotional stimuli produce worse inhibition capacity compared to neutral stimuli (i.e., longer SSRT; Song et al., 2016; Ding et al., 2020), while an SST with task-irrelevant emotional stimuli yielded varied outcomes with respect to inhibitory performance (Sagaspe et al., 2011; Pawliczek et al., 2013; Rebetez et al., 2015; Derntl and Habel, 2017). Emotional stimuli presented as primes have been reported to interfere with the action control capabilities, lengthening the SSRT (Verbruggen and Houwer, 2007; Krypotos et al., 2011; Kalanthroff et al., 2013). On the other hand, emotional stimuli presented as stop signals were found to facilitate action control (Pessoa et al., 2012; Senderecka, 2016, 2018). Similarly, in our recent works (Battaglia et al., 2022a,b), we demonstrated that emotional stimuli presented as stop signals can increase action inhibition capabilities (i.e., shorter SSRT) relative to neutral control stimuli. Overall, it seems that the detrimental effect obtained from presenting task-relevant emotional stimuli (Calbi et al., 2022; Mancini et al., 2022) or task-irrelevant emotional stimuli as primes can be elucidated through the attentional account proposed by Schimmack (2005), suggesting that emotional stimuli attract attention and consequently disrupt the execution of the ongoing task (Pessoa, 2009; Pessoa et al., 2012). A way to test the attentional account is to investigate whether presenting subliminal task-irrelevant emotional stimuli as a prime is still able to impact high cognitive processes, such as action control. A significant portion of cognitive processes can transpire non-consciously and influence behavior (Merikle et al., 2001; Eimer and Schlaghecken, 2003; Kunde, 2003; Lau and Passingham, 2007). In particular, there is now considerable evidence that the processing of potentially dangerous stimuli can occur even outside conscious awareness (Morris et al., 1998; Whalen et al., 1998; Diano et al., 2017). Behavioral studies have provided evidence that undetected fearful faces and bodies can influence the assessment of a subsequent visible probe stimulus (Yang et al., 2011). This influence extends to various cognitive processes, such as, among others, the orienting of covert spatial attention (Carlson and Reinke, 2008) as well as the recognition of happy faces (Tamietto and de Gelder, 2008). Interestingly, the influence of non-conscious stimuli on motor responses has already been investigated (Engelen et al., 2018) whereas the role of emotion awareness in the control of action remains unresolved. Here, we investigated this issue by presenting our participants with a modified version of our SST (Battaglia et al., 2022b) in which the same emotional (i.e., fearful) and neutral body postures previously presented as stop signals were now briefly flashed (~17 ms) and sandwich-masked before the go signals. If emotionally salient negative stimuli, even though non-consciously presented, are able to impede action control, we should expect longer SSRT in trials with negative stimuli compared to neutral stimuli presented as primes. Contrary to this idea, the fact that the negative stimuli are presented subliminally may enhance action control (i.e., faster SSRT), is consistent with the freezing account, which suggests that emotional stimuli might lead to a temporary suspension of all task-unrelated ongoing activities, thereby enhancing action control (Ohman et al., 2001; Flykt, 2006).

Moreover, prior studies employing Transcranial Magnetic Stimulation (TMS) during the SST indicate that the suppression of a motor response relies on contextual modulation of corticospinal excitability and intracortical inhibition within the primary motor cortex (M1; Duque et al., 2017). While motor-evoked potentials (MEPs) provide a reliable measure of corticospinal excitability, paired-pulse TMS is administered to directly assess modulations of intracortical excitability within M1. To do this, a conditioning TMS pulse below the threshold intensity needed to elicit an MEP is followed at short interstimulus intervals (ISIs) by a suprathreshold test TMS pulse eliciting a MEP. Certainly, at ISIs of 1–5 ms, the conditioning pulse induces a reduction in the MEP elicited by the test pulse, denoted as short intracortical inhibition (SICI). Conversely, longer ISIs in the range of 7–20 milliseconds lead to MEP facilitation, recognized as intracortical facilitation (ICF). Specifically, it is theorized that SICI and ICF primarily represent the activation of low-threshold inhibitory interneurons mediated by gamma-aminobutyric acid (GABA) (Ziemann et al., 1996; Di Lazzaro et al., 2000; Ilic et al., 2002) and glutamatergic interneurons (Nakamura et al., 1997; Ziemann, 2003), respectively. Notably, SICI and ICF are modulated by the observation of emotionally relevant stimuli (Borgomaneri et al., 2015b,c, 2017) and interindividual differences in SICI predict better action suppression (He et al., 2019; Chowdhury et al., 2019a,b; Tran et al., 2020; Ding et al., 2021; Loomes et al., 2023). Therefore, here we additionally tested whether action control in the context of subliminally presented emotional stimuli can be influenced by individual differences in resting-state intracortical measures of motor excitability. Our investigation aims to provide new insights into the intricate relationship between emotions, consciousness, and action control, suggesting potentially useful biomarkers for assessing deficits in action control in the psychiatric domain, in which several pathologies suffer deficits in action control in an emotional context (Battaglia et al., 2021; Di Gregorio and Battaglia, 2024).

## Materials and methods

### Participants

In this study, 46 right-handed, healthy individuals with normal or corrected-to-normal vision participated. All participants completed the SST. We organized the experiment into two distinct groups. The first group, comprising 30 participants [Behavioral Group; mean age = 23.43, standard deviation (S.D.) = 3.32, 16 females] completed our previously published modified version of the SST (Battaglia et al., 2022b). The second group (Neurophysiological Group; mean age = 23.60, S.D. = 2.10, 12 females) included 20 participants. Four participants in the Neurophysiological group were excluded because of technical failures either in the SST or in the neurophysiological recording. Participants in this group performed the same SST as the Behavioral Group and additionally underwent neurophysiological measurements, such as SICI and ICF. All participants were confirmed as right-handed using the Edinburgh Handedness Inventory (Oldfield, 1971). Participants were uninformed about the objectives of the experiment and reported no history of neurological or psychiatric disorders, visual impairments, medication usage, or any contraindications to TMS (Rossi et al., 2021). The sample size for the Behavioral group was determined through power analysis, revealing that a total of 30 participants is required to achieve a statistical power (1−β) of 0.99 (two-tailed α = 0.01; effect size *f* = 0.40) (similar effect sizes were reported in studies such as Verbruggen and Houwer, 2007; Krypotos et al., 2011) number of measurements = 2; correlation = 0.5, analysis performed with G*Power software (Faul et al., 2007). Another power analysis determined that a sample size of 20 participants is required to attain a statistical power (1−β) of 0.90 (two-tailed *α* = 0.05; *r* = 0.65) in the Neurophysiological group (see for example Chowdhury et al., 2018; Loomes et al., 2023). The two groups were matched for age [*t*(42) = −0.08, *p* = 0.93; *d* = 0.03] and gender [χ^2^ (1, *N* = 46) = 0.03, *p* = 0.86]. Furthermore, considering the observed impact of personality traits such as trait anxiety (Avila and Parcet, 2001; Neo et al., 2011; Toh and Yang, 2020; Hsieh et al., 2022) and impulsivity (Logan et al., 1997; Avila and Parcet, 2001; Bari and Robbins, 2013; Pawliczek et al., 2013) on the inhibitory control, we conducted an additional examination. Specifically, we investigated whether these personality traits play a role in influencing action control when emotionally negative stimuli are presented as primes (i.e., before the Go stimulus). Subjective anxiety levels were assessed using the State–Trait Anxiety Inventory (STAI; Trait-scale-Y2) (Spielberger et al., 1970) while subjective impulsivity levels were measured with the Barratt Impulsiveness Scale-11 (BIS-11) (Patton et al., 1995). The STAI-Y2 comprises a 20-item self-report questionnaire that gages anxiety frequency. The BIS-11 is a self-report questionnaire with 30 items, evaluating both impulsive and non-impulsive behaviors. The two groups did not show any significant difference in terms of anxiety [STAI-Y2: *t*(44) = 0.09, *p* = 0.92, *d* = 0.03] but a difference was found in terms of impulsivity scores [BIS-11: *t*(44) = 3.57, *p* < 0.01, *d* = 1.08] in which the Behavioral Group showed a higher score than the Neurophysiological Group (Behavioral Group: mean = 70.20, S.D. = 7.20; Neurophysiological Group: mean = 62.60, S.D. = 6.19). However, such a difference did not affect our findings (see the Results section).

Data collection was conducted anonymously, with all participants providing their informed consent before engaging in the task. The study was conducted in accordance with the ethical principles of the World Medical Association Declaration of Helsinki and was approved by the Bioethical Committee of the University of Bologna.

### Experimental procedure

In this study, all participants engaged in the same behavioral task followed by the awareness questionnaires and the assessment of personality traits. Only the Neurophysiological Group underwent an additional TMS session (at the beginning of the experimental session), in which neurophysiological parameters such as single-pulse MEPs, SICI, and ICF were measured at rest, to assess corticospinal excitability, short-interval intracortical inhibition, and intracortical facilitation, respectively.

**Figure 1:**
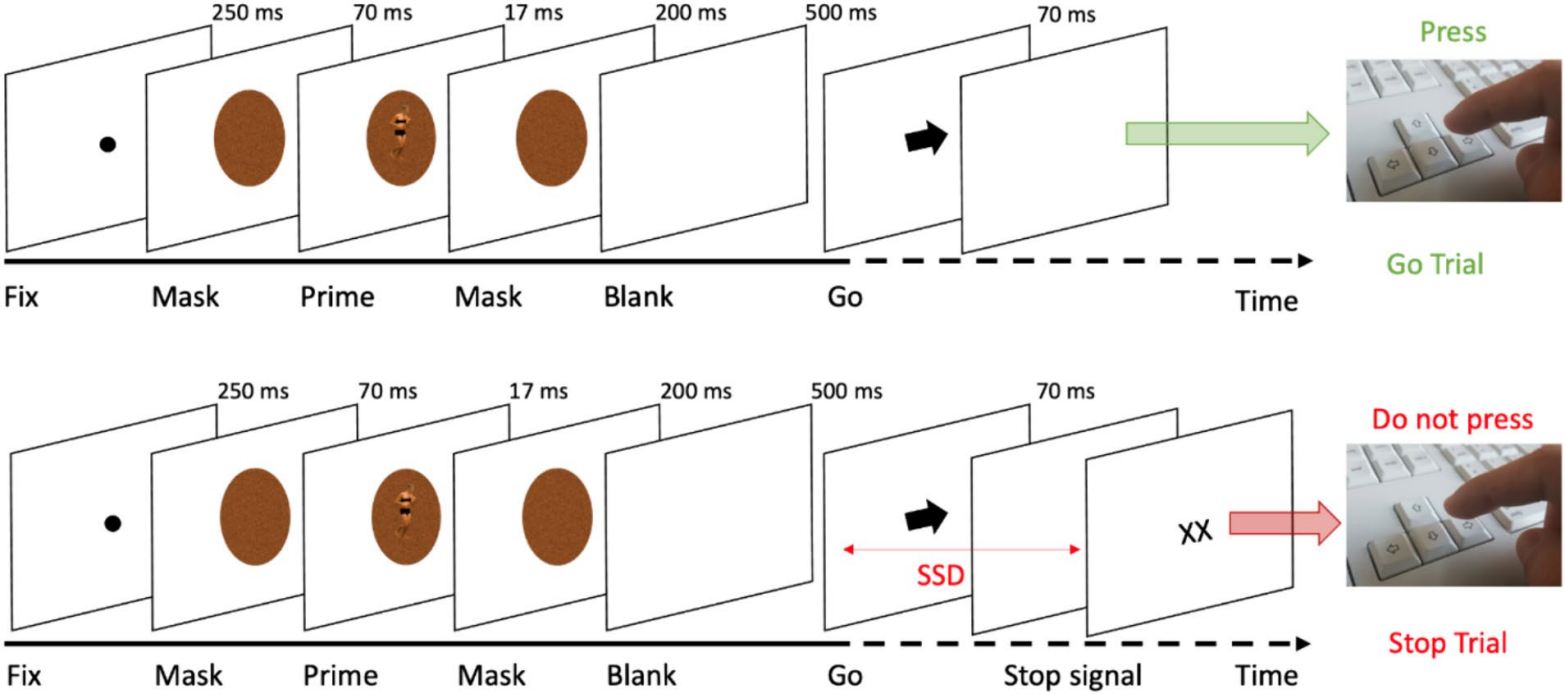
Trial sequence in the SST in Go (top) and Stop-trials (bottom).

The SST required participants to execute a basic reaction time (RT) task, incorporating both Go and Stop-trials, a methodology grounded in foundational work (Logan, 1994; Verbruggen and Logan, 2008). Typically, in SSTs, participants respond to Go stimuli (e.g., pressing left for a left-pointing arrow, right for a right-pointing arrow) but must inhibit their response when a Stop signal, indicated here as “XX,” appears following a variable delay (see Figure 1).

**Figure 2:**
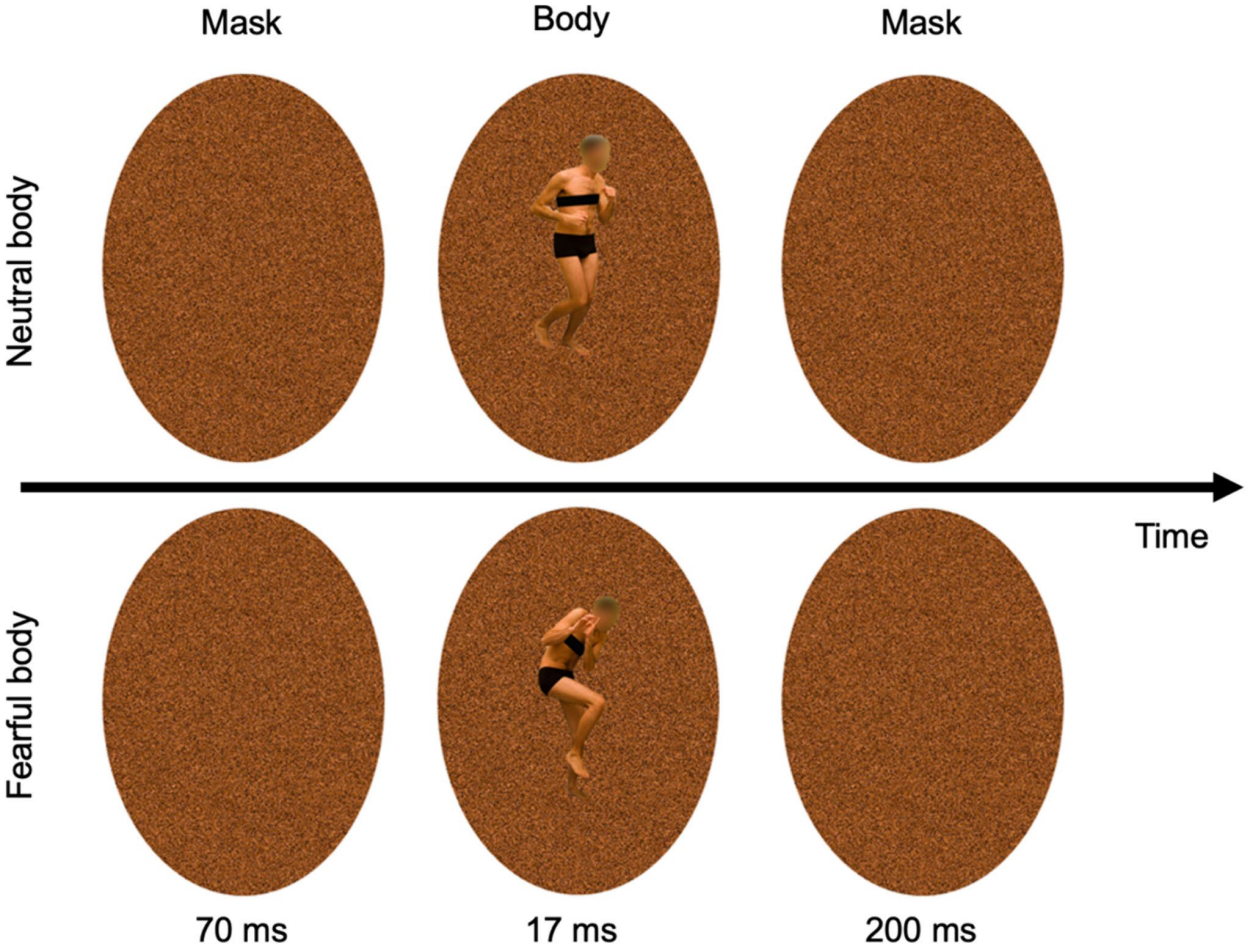
Masks and prime stimuli (neutral and fearful body postures).

The task commenced with a preliminary practice block of 32 trials, succeeded by four experimental blocks. Each block consisted of 64 trials, with a distribution of 75% Go-trials (48 trials) and 25% Stop-trials (16 trials), cumulatively amounting to 256 trials. Every trial began with a black dot centered on a white screen, serving as a fixation point for 250 ms. This was followed by a noise-like pattern mask (see Figure 2), displayed for 70 ms, created using custom image segmentation software. After this mask, an image of a body (either expressing fear or neutral emotion) was flashed briefly (~17 ms) and was immediately replaced by the same mask stimulus (200 ms). The body postures, sourced from a validated database ensured that arousal, valence, and implied motion were matched (Borgomaneri et al., 2012, 2015a,b, 2017, 2020a,c, 2024), were presented in both Go and Stop-trials as prime stimuli. After the second mask, a blank screen appeared for 500 ms before the presentation of the Go signal (i.e., the arrow). In Go-trials, participants responded quickly and accurately to the arrow direction, visible for 70 ms. After the Go signal, a blank screen appeared until the end of the trial. The trial ended at the participant’s response or 1,500 ms after the Go signal. Conversely, in Stop-trials, participants were instructed to withhold their response upon the appearance of the Stop signal, displayed until the participant’s response or 1,500 ms after the Go signal, following a variable Stop-signal delay (SSD; i.e., the time between the go and the stop signals presentation) with respect to the Go signal onset. The initial SSD was set at 250 ms, and was dynamically adjusted for each trial using a staircase procedure (Band et al., 2003; Matzke et al., 2018; Verbruggen et al., 2019), targeting a 50% success rate in Stop-trials. Importantly, separate staircases were computed for each specific condition (fearful and neutral prime stimuli). The staircase was independent within-subject, as the SSD was adjusted individually based on performance in 50 ms increments (ranging from 50 to 650 ms), separately for emotional and neutral prime trials. In particular, if participants successfully inhibited their response on a Stop-trial, the SSD was increased by 50 ms on a subsequent Stop-trial, while if they failed to withhold their motor response, the SSD was reduced by 50 ms on a subsequent Stop-trial. Finally, the next trial was presented after a 500 ms interval. Participants were instructed to respond as quickly and accurately as possible to the arrow and were asked to inhibit their response upon viewing a stimulus that followed the initial Go-signal that appeared on the screen. However, they were also instructed that sometimes it might not be possible to successfully inhibit their response and, in such cases, they should continue to perform the task irrespective of having made an error (Pessoa et al., 2012; Verbruggen et al., 2019). Moreover, participants were instructed not to hesitate or decelerate in order to minimize the likelihood of stopping. In general, our SST was crafted in accordance with the guidelines provided by Verbruggen et al. (2019). After the task, we assessed participants’ abilities to process the 17 ms prime bodies. We evaluated subjective awareness of the prime bodies using targeted questions of the priming phase (Sweeny et al., 2009). The participants were asked to respond to the following questions: (1) “Did you see anything other than the arrow and the crosses?” (2) “Was there a stimulus just before the arrow, and if so, could you identify what it was?” (3) “Did you see a body?” (4) “What posture did it have?” A report acknowledging awareness of the presence of a body in a neutral (i.e., running) and a negative (i.e., fearful) posture was considered as an indication that the prime presence was perceived. Upon completing the subjective awareness questionnaire, participants were then asked to fill out the personality traits questionnaires.

### TMS and electromyography recording

Following the completion of the behavioral tasks, the Neurophysiological Group underwent the electrode montage setup, detection of optimal scalp position, and measurement of resting motor threshold (rMT). To investigate motor excitability, MEPs generated by TMS applied to the left M1 were captured from the right first dorsal interosseus (FDI) muscle. A Biopac MP-35 (Biopac, United States) electromyograph, was utilized for recording. The electromyogram (EMG) signals underwent band-pass filtering (30–500 Hz), were sampled at a rate of 5 kHz, digitized, and then stored on a computer for subsequent offline analysis. A belly-tendon montage was used, and pairs of silver-chloride surface electrodes were positioned, with ground electrodes on the wrist. A figure-of-eight coil, connected to a Magstim Bistim2 stimulator (Magstim, Whitland, Dyfed, United Kingdom), was then positioned over the M1. The intersection of the coil was placed tangentially to the scalp with the handle pointing backward and laterally at a 45° angle away from the midline. In this way, the current induced in the neural tissue was directed approximately perpendicular to the line of the central sulcus, optimal for trans-synaptic activation of the corticospinal pathways (Brasil-Neto et al., 1992; Mills et al., 1992). Employing a slightly suprathreshold stimulus intensity, the coil was moved over the left hemisphere to identify the optimal position that elicited maximal MEPs in the contralateral FDI muscle. Subsequently, the identified optimal position of the coil was marked on the scalp with a pen to maintain accurate coil placement throughout the experiment. The rMT was defined as the minimal intensity of stimulator output that generated MEPs with an amplitude of at least 50 μV with 50% probability, determined by approximately 20 pulses (Rossini et al., 1994). The absence of voluntary contraction was visually confirmed throughout the experiment. If muscle tension was observed, the experiment was momentarily paused, and the subject was instructed to relax.

Motor-evoked potentials were recorded in three sessions: Single pulse (SP), SICI, ICF. During the SP session, intensity was set to evoke MEPs with a peak-to-peak amplitude of ~1.0 mV. During the paired-pulse TMS paradigm, SICI and ICF were measured using an established protocol (Kujirai et al., 1993; Ziemann et al., 1996). The conditioning (CS) and test (TS) stimuli were administered using the same coil. The intensity of the CS was set at 80% of the rMT, a level at which MEPs were consistently not induced. The TS intensity matched that used in the SP session. Two ISIs, specifically 3 and 12 ms, were chosen, as these are commonly employed for investigating SICI and ICF circuits, respectively (Kujirai et al., 1993; Ziemann et al., 1996; Borgomaneri et al., 2015a,b, 2017).

### Data processing and analysis

In this investigation, reactive inhibition indices were computed for each specific condition (fearful and neutral prime stimuli) within the SST, employing the SSRT measurement, as delineated in prior work by Battaglia et al. (2022b), and in alignment with the race model concept proposed by Logan et al. (1984). Before delving into SSRT analysis, we confirmed the reliability of participant performance on the SST by assessing the inhibition rate, which is expected to hover around the 50% mark, as per the guidelines of Band et al. (2003), Logan et al. (2014), Matzke et al. (2018), and Verbruggen et al. (2019). Adhering to the methodology advocated by Verbruggen et al. (2013), our SSRT estimation employed the integration method, incorporating Go-trial omissions into the calculations. This method involves integrating the distribution of RTs from Go-trials to pinpoint the moment when the cumulative distribution matches the probability of responding post-stop-signal presentation, denoted as “p(respond| signal).” The conclusion of the stopping process is indicated at the point on the RT distribution curve where the integral equals this probability. Specifically, the ending time of the stop process corresponds to the *n*th RT, where *n* equals the total number of RTs in the Go-trial RT distribution multiplied by “p(respond|signal).” For determining the *n*th RT, all responses in Go-trials are considered, including those with choice errors and premature responses. It is crucial to note that omissions (i.e., Go-trials where participants did not respond before the trial’s end) are assigned the maximum RT to account for the absence of a response. Furthermore, premature responses in unsuccessful Stop-trials (responses executed before the presentation of the Stop-signal) are included in the calculation of “p(respond| signal)” and the mean SSD. This approach, known as the integration method, is recognized for producing the most reliable and least biased estimation of the SSRT (for a comprehensive review and detailed explanation of this methodology, see Verbruggen et al., 2019). For the analysis, we utilized custom scripts developed in MATLAB (The MathWorks, Inc., Natick, MA, United States) to estimate SSRT, and conducted the statistical analyses using R software (R Foundation for Statistical Computing, Vienna, Austria). Our approach included ANOVAs to investigate the effect of the Prime stimulus (Fearful/Neutral) as a within-subject factor and the Group (Behavioral/Neurophysiological) as a between-subject factor. *Post hoc* analyses were conducted with Bonferroni test and the significance threshold was set at *p* < 0.05. Mean MEP amplitudes were measured peak-to-peak (in mV). Since background EMG is known to affect motor excitability (Devanne et al., 1997), MEPs were visually inspected and the ones preceded by background EMG were removed from further analysis. To measure the effects of ICF and SICI, we normalized MEPs in the paired-pulse sessions by comparing them to the SP session. This involved estimating the impact of the subthreshold CS on the MEP elicited by the suprathreshold TS. The ratio was then calculated by dividing the mean conditioned MEP by the mean unconditioned test MEP (Kujirai et al., 1993; Ziemann et al., 1996).

## Results

### Verification of the SST assumptions

We commenced our analysis by examining the foundational assumptions of the independent race model as outlined by Verbruggen et al. (2019). Our primary focus was to compare whether the mean RT during Unsuccessful Stop-trials (instances where participants failed to halt their action despite a Stop-signal) was shorter than the mean RT in Go-trials. In our analysis of RTs, we conducted a 2 × 2 ANOVA with Trial type (Go/Unsuccessful Stop) as a within-subject factor and Group (Behavioral/Neurophysiological) as a between-subject factor, aimed at exploring the processing differences between these trial types. The analysis revealed a main effect of Trial type [*F*(1,44) = 47.87, *p* < 0.01, ηp^2^ = 0.96], with significantly longer RTs for Go-trials (mean = 549 ms, S.D. = 18.2 ms) compared to Unsuccessful Stop-trials (mean = 482 ms, S.D. = 13.4 ms). The consistency of longer RTs for Go-trials aligns with the theoretical expectations of the SST, where Go-trials typically require more cognitive processing (see Table 1 for details). Next, we validated the efficacy of the staircase procedure. Our goal was to confirm that the inhibition rate (the proportion of successful stops when a Stop-signal is presented) hovered around the 50% mark across all priming, as detailed in Table 1. We then proceeded to conduct a 2 × 2 ANOVA on the inhibition rate, considering the Prime (Fearful/Neutral) as a within-subject factor and the Group (Behavioral/Neurophysiological) as a between-subject factor. The findings indicated no significant differences in inhibition rate across Groups [*F*(1,44) = 0.054, *p* = 0.81, ηp^2^ = 0.016] or Primes [*F*(1,44) = 0.08, *p* = 0.77, ηp^2^ = 0.025]. Furthermore, the Group × Prime interaction was also non-significant [*F*(1,44) = 3.05, *p* = 0.08, ηp^2^ = 0.95], suggesting a uniform stop performance percentage when a Stop-signal is presented, irrespective of prime stimuli and participant groups.

**Table 1:** Behavioral data collected in the SST.

Group	Behavioral		Neurophysiological	
SST	Fearful body	Neutral body	Fearful body	Neutral body
Inhibition rate (%)	52.82 ± 10.64	54.16 ± 11.11	54.68 ± 8.54	53.71 ± 7.76
SSD (ms)	263.44 ± 105.27	249.17 ± 104.19	216.99 ± 42.08	205.96 ± 36.89
SSRT (ms)	256.72 ± 102.76	336.02 ± 44.54	326.82 ± 30.06	334.9 ± 32.75
Unsucc RT (ms)	491.15 ± 102.65	496.15 ± 106.51	474.71 ± 38.68	466.6 ± 43.24
Go RT (m)	568.05 ± 140.61		529 ± 46.72	
Correct Go (%)	98.77 ± 1.43		99.22 ± 1.64	

The descriptive performance of the SST is reported as means ± S.Ds. In particular, inhibition rate, stop signal delay (SSD), stop signal reaction time (SSRT), unsuccessful reaction time (Unsucc RT), go reaction time (Go RT), and correct go responses are depicted in the table for each group.

We extended our investigation to the proportion of correct responses in Go-trials across groups, using a similar 2 × 2 ANOVA with Prime (Fearful/Neutral) and Group (Behavioral/Neurophysiological) factors. Results showed no significant Group differences [*F*(1,44) = 0.92, *p* = 0.34, ηp^2^ = 0.15], nor any influence of the factor Prime [*F*(1,44) = 3.38, *p* = 0.07, ηp^2^ = 0.57]. The interaction effect was also not significant [*F*(1,44) = 1.57, *p* = 0.21, ηp^2^ = 0.26], indicating consistent performance accuracy across all groups and prime stimuli. Additionally, we analyzed the Go-trial RTs with a 2 × 2 ANOVA with Prime (Fearful/Neutral) and Group (Behavioral/Neurophysiological) as factors. The analysis did not reveal any significant differences in reaction times between Groups [*F*(1,44) = 0.18, *p* = 0.28, ηp^2^ = 0.08] nor were they influenced by the factor Prime [*F*(1,44) = 0.54, *p* = 0.96, ηp^2^ = 0.23]. A significant Prime × Group interaction was observed [*F*(1,44) = 1.57, *p* = 0.03, ηp^2^ = 0.68]. However, Bonferroni *post hoc* comparisons revealed no significant results (all *p* > 0.5; RT Behavioral: mean Fear = 564 ms, S.D. = 22 ms, mean Neutral = 572 ms, S.D. = 21 ms; RT Neurophysiological: mean Fear = 533 ms, S.D. = 30 ms, mean Neutral = 525 ms, S.D. = 28 ms). Results suggest homogeneous reaction times across different conditions. Lastly, we analyzed the SSD data through a 2 × 2 ANOVA with Prime (Fearful/Neutral) and Group (Behavioral/Neurophysiological) factors. The results highlighted no significant Group differences [*F*(1,44) = 2.79, *p* = 0.1, ηp^2^ = 0.29]. Interestingly, a significant impact of the factor Prime [*F*(1,44) = 6.58, *p* = 0.01, ηp^2^ = 0.69] was found with a 10 ms shorter SSD for Neutral primes (mean = 228, S.D. = 13.5) than for Fearful primes (mean = 240, S.D. = 13.8). As expected, the emotional content of the Prime stimuli impacted the execution of participants’ actions, resulting in a distinct differentiation of SSD that was appropriately adjusted through successful staircase procedures. Additionally, the interaction among these factors was not significant [*F*(1,44) = 0.107, *p* = 0.74, ηp^2^ = 0.01], indicating a uniform SSD across all participant groups and primes.

In conclusion, these analyses confirm the reliability of the SST data collected during our experimental phases. The foundational assumption of an appropriate inhibition rate stands validated, paving the way for a reliable estimation of the SSRT, in line with Verbruggen et al. (2019).

### The negative emotional content of stimuli boosts the ability to suppress an ongoing action

**Figure 3:**
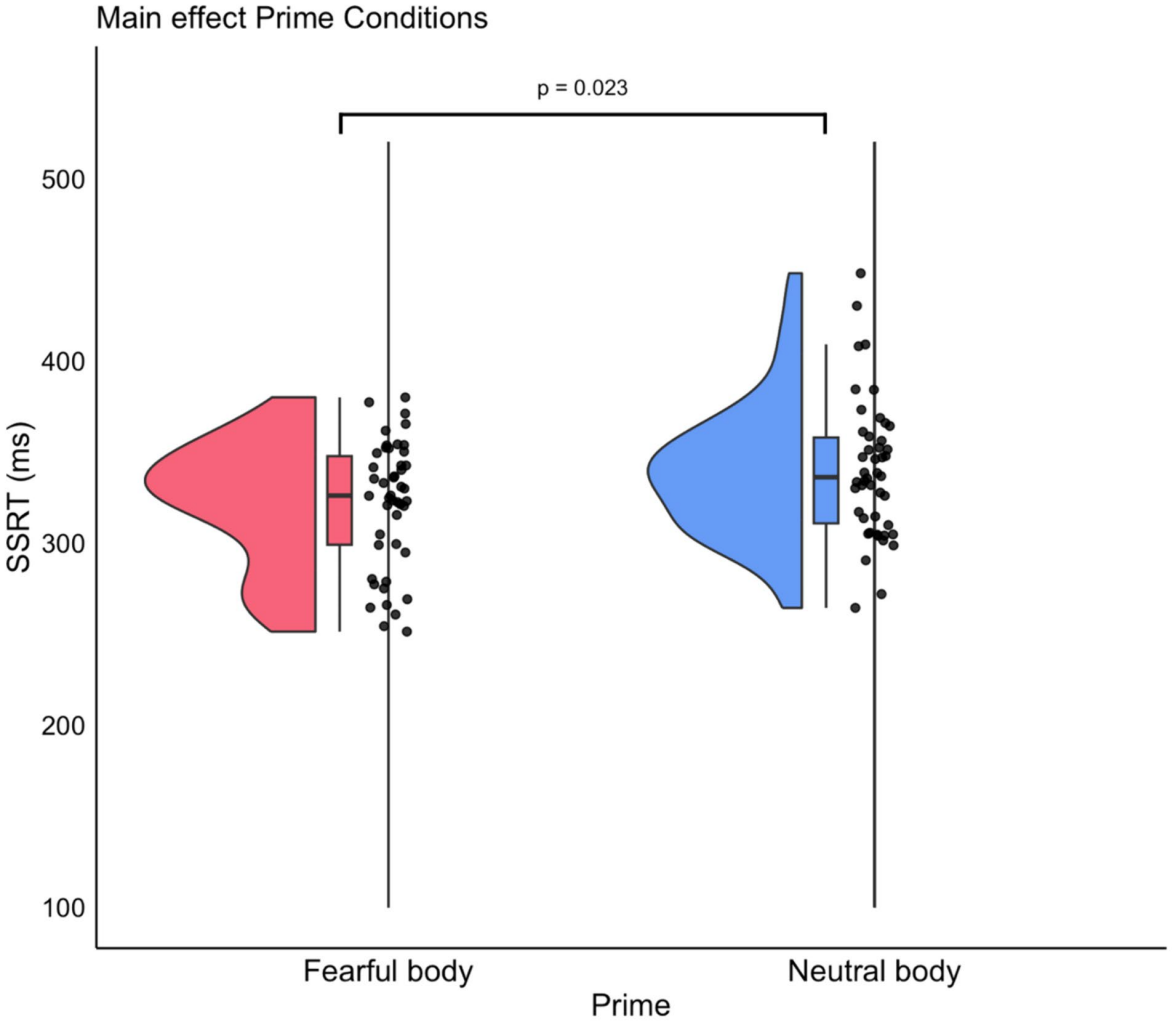
*Post hoc* comparison of the SSRT between prime conditions. The violin plot illustrates the distribution of the SSRT across the two prime conditions: Fearful and Neutral body postures presentation. Each violin shape represents the density distribution of the SSRT values, with wider sections indicating a higher concentration of data points. Boxplots summarize the median (central line), interquartile range (box edges), and range excluding outliers (whiskers) of SSRTs within each prime condition.

The analysis of the SSRT was complemented by a 2 × 2 ANOVA with Prime (Fearful/Neutral) as a within-subject factor and Group (Behavioral/Neurophysiological) as a between-subject factor. The ANOVA results indicated a significant main effect of the factor Prime on SSRT [*F*(1, 44) = 5.56, *p* = 0.02, ηp^2^ = 0.81] in which SSRT related to Fear prime stimuli are shorter than those for Neutral prime stimuli (Fearful: mean = 322, S.D. = 5.91; Neutral: mean = 338, S.D. = 5.91) (see Figure 3). As expected, no main effect for the Group factor was found [*F*(1, 44) = 0.01, *p* = 0.9, ηp^2^ = 0.002]. This suggests a similar response inhibition ability across groups. The Prime × Group interaction was also found to be non-significant [*F*(1, 44) = 1.25, *p* = 0.27, ηp^2^ = 0.18]. Out of 46 participants, two explicitly reported the presence of the neutral and fearful body postures as primes. Thus, we carried out the same Prime × Group ANOVA removing these two participants to ensure that the results were not influenced by this factor. The ANOVA results confirmed the main effect of the Prime factor [*F*(1, 42) = 4.78, *p* = 0.03 ηp^2^ = 0.79], while the main effect of the factor Group was not found to be significant [*F*(1, 42) = 0.07, *p* = 0.79, ηp^2^ = 0.01], nor was the Prime × Group interaction [*F*(1, 42) = 1.13, *p* = 0.29, ηp^2^ = 0.18]. These results are in line with previous findings, which demonstrate that action control is influenced by the presence of emotional stimuli (see Battaglia et al., 2021 for a review on the topic) but, crucially, our findings demonstrated that such effects are detectable even when the negative arousing stimuli are not consciously perceived, corroborating to the idea that consciousness of the emotional stimuli is not a prerequisite to observe an influence on behavior. To assess whether differences in participants’ impulsivity may have an impact on the results, we conducted an analysis using a Generalized Linear Mixed Model with Prime, BIS-11, and their interaction as fixed effects and subject as a random effect. Model comparisons revealed a significant improvement in fit when the factor Prime was included as a predictor [AIC = 924.39, BIC = 934.48, χ^2^(4) = 7.51, *p* < 0.01]. However, the further inclusion of BIS-11 scores was also significant [AIC = 925.86, BIC = 938.47, χ^2^(6) = 7.51, *p* < 0.01]. Therefore, considering both statistical significance and goodness-of-fit measures, a model including only the Prime factor is the best balance between explanatory power and simplicity.

### Relation between changes in action control and motor excitability

**Figure 4:**
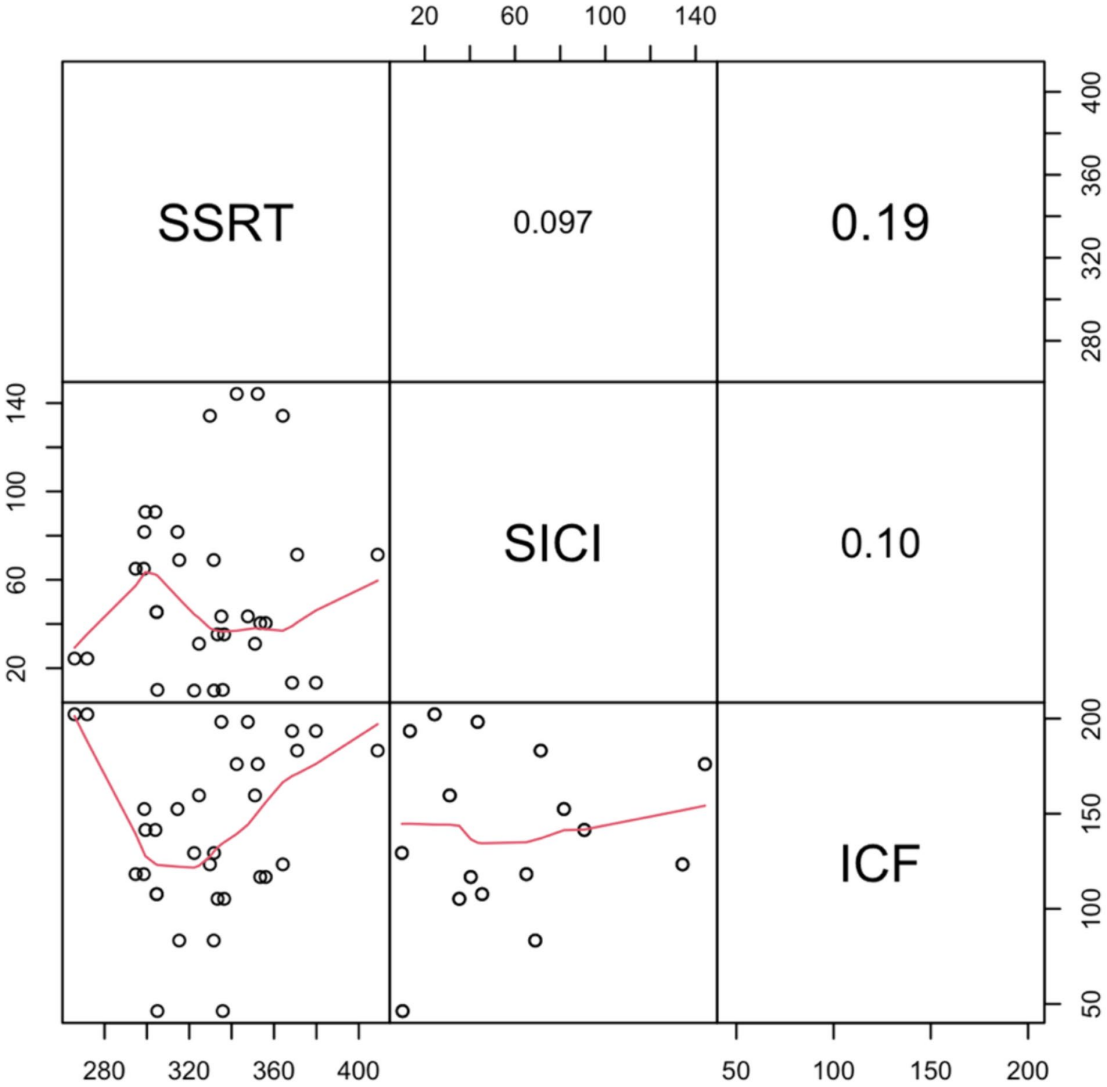
Correlation matrix of Stop Signal Reaction Times (SSRT) with neurophysiological indices (Short Intracortical Inhibition—SICI and Intracortical Facilitation—ICF). The off-diagonal scatter plots with red trend lines depict the pairwise correlations. Significance levels are indicated as follows: . denotes *p* < 0.1; ^*^p < 0.05; ^**^p < 0.01; and ^***^p < 0.001.

To investigate the relationship between the SSRT and neurophysiological measures, regression analyses were conducted. In a stepwise regression model, with SSRT as the dependent variable, SICI and ICF were introduced as predictors. Initially, we examined SSRT as an index calculated as the difference between the two prime stimuli (neutral minus fearful). This regression model was significant [*R*^2^ = 0.27; *F*(1,14) = 5.19, *p* = 0.04]. This finding is in line with previous results (He et al., 2019; Loomes et al., 2023) suggesting that lower levels of SICI correspond to an inhibition advantage. Additionally, here we have demonstrated that such behavioral advantage is unrelated to the prime stimulus that was (subliminally) presented. Moreover, we decided to investigate whether individual SICI and ICF may predict action control performance regardless of the type of prime presented. To do so, we evaluated SSRT by averaging the SSRTs related to the observation of the neutral and the fearful prime stimuli. In this case, the regression model including SICI as a predictor did not yield any significant results [*R*^2^ = 0.01; *F*(1,14) = 0.14, *p* = 0.71]. No predictor showed a significant positive correlation with SSRT in this context (Figure 4).

### Relation between changes in action control and personality

**Figure 5:**
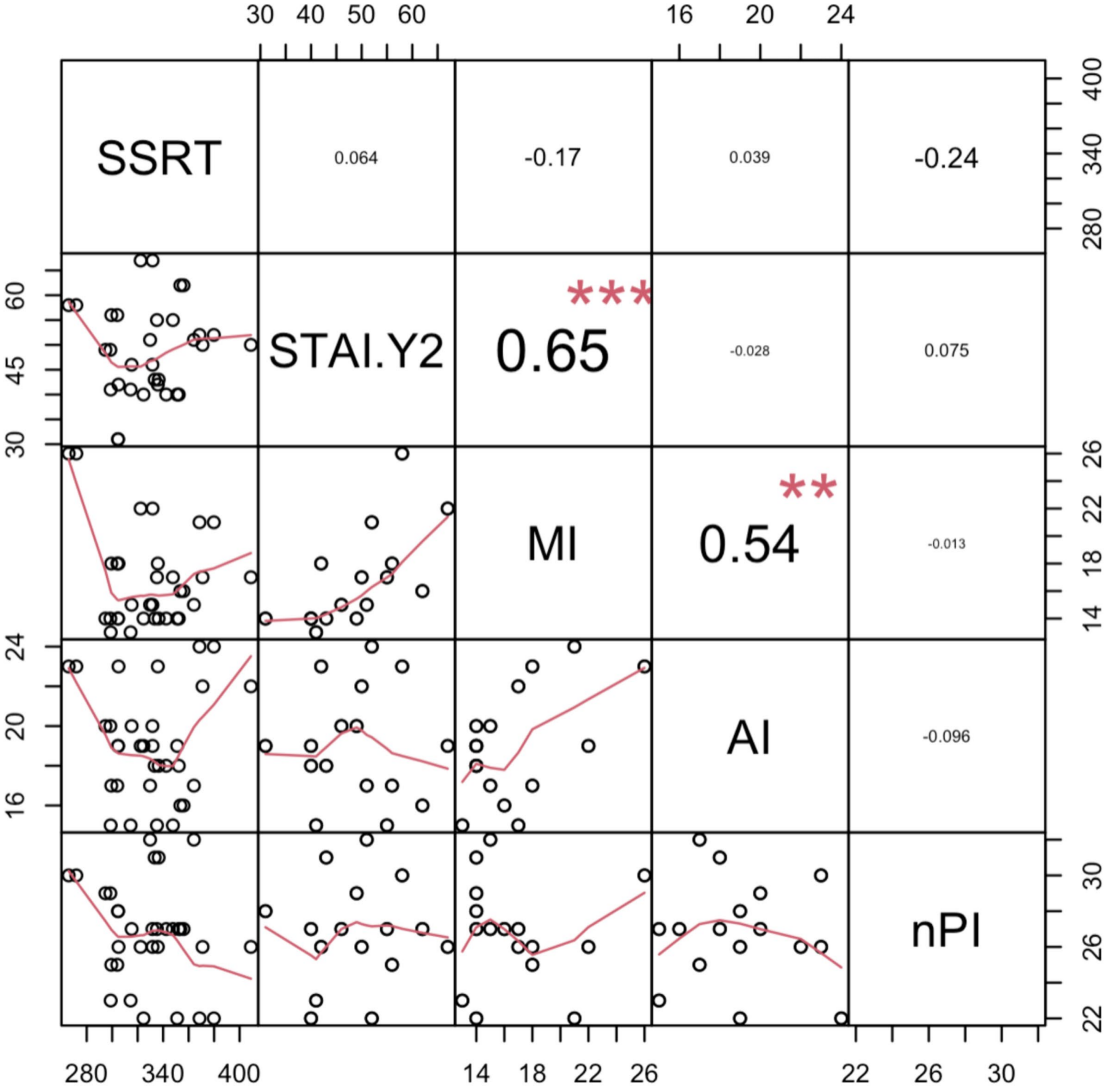
Correlation matrix of SSRT with personality traits questionnaires. The off-diagonal scatter plots with red trend lines depict the pairwise correlations. In particular, Stop Signal Reaction Times (SSRT), State-Trait Anxiety Inventory (STAI), and all the Barratt Impulsiveness Scale-11 subscale: MI, Motor impulsivity; AI, Attentional impulsivity; and nPI, Non-planning impulsivity. Significance levels are indicated as follows: . denotes *p* < 0.1; ^*^p < 0.05; ^**^p < 0.01; and ^***^p < 0.001.

In exploring the relationship between SSRT and personality traits, correlation and regression analyses were carried out using the full participant sample. An SSRT index calculated as the difference between the two prime stimuli (neutral minus fearful), was entered as the dependent variable in a stepwise regression model, with the STAI-Y2 and BIS-11 subscales (i.e., MI: motor impulsivity; AI: attentional impulsivity; nPI: non-planning impulsivity) entered as predictors (Battaglia et al., 2022b). This analysis results in a significant regression model [*R*^2^ = 0.11; *F*(1,43) = 4.56, *p* = 0.04]. However, after removing two statistical outliers with a residual greater than 2 sigma, the model was non-significant [*R*^2^ = 0.02; *F*(1,41) = 2.02, *p* = 0.16], and no predictor showed a significant correlation with SSRT in this context. Next, an SSRT index calculated as the mean between the two prime stimuli (averaging neutral and fearful) was used as the dependent variable. Again, the regression model was non-significant [*R*^2^ = 0.0003; *F*(1,44) = 0.016, *p* = 0.9], even after the removal of three statistical outliers with residuals greater than 2 sigma [*R*^2^ = 0.0002; *F*(1,44) = 0.01, *p* = 0.92], with no predictor showing a significant correlation with SSRT (see Figure 5). These findings demonstrated that personality traits such as impulsivity and anxiety did not influence our findings.

## Discussion

Perceiving negative salient cues will likely trigger an adequate motor response in the observer, aligning with the idea that emotion and action readiness are closely interconnected (Frijda, 2010; Borgomaneri et al., 2014, 2021). Overall, akin to consciously perceived threats, it appears that non-consciously perceived threats may also be linked to action preparation, as indicated, for instance, by changes in heart rate (Ruiz-Padial et al., 2005, 2011). However, it was still unclear whether non-consciously perceived threats may influence action control capabilities, measured using the SST, which can offer a reliable measure of the time taken by the brain to cancel an ongoing action (i.e., SSRT). By presenting subliminal task-irrelevant negative or neutral prime stimuli before the go signal, we investigated their influence on the ability to stop the participants’ ongoing action (i.e., discriminating the orientation of the go arrow signal) when a neutral stop stimulus (i.e., a series of crosses) was presented. Additionally, we aimed to test whether individual measures of SICI and ICF can be used as neurophysiological markers to predict action cancelation performance when subliminal emotional stimuli are presented. Results demonstrated that participants showed better action control in trials in which fearful task-irrelevant body expressions were presented compared to neutral body posture presentation. The current findings reinforce the idea that the perception of emotion is inherently connected to action systems, and provide additional evidence for the existence of a “negative bias” also for non-consciously presented threatening stimuli, shedding new light on the way non-conscious negative stimuli impact higher cognitive functions, such as action control.

The majority of current studies utilizing the SST with emotional stimuli have consistently shown that presenting an emotional image before the go stimulus tends to hinder the ability to inhibit an action (Verbruggen and Houwer, 2007; Kalanthroff et al., 2013; Rebetez et al., 2015). Conversely, when the emotional stimulus serves as the stop signal, a facilitatory effect is generally observed (Pessoa et al., 2012; Senderecka, 2016, 2018). Moreover, some studies using go/no-go tasks found that emotional stimuli impacted action control only when task-relevant, but not when task-irrelevant (Calbi et al., 2022; Mancini et al., 2022). However, go/no-go tasks and SST recruit widely different neural dynamics (Raud et al., 2020), and the abovementioned studies did not employ subliminal stimuli, but rather the emotion conveyed by the stimulus is relevant to the task.

Importantly, while impulsivity is found to impact the ability to halt an action in response to negative stimuli acting as a stop signal (Battaglia et al., 2022b), this is no longer applicable in the context of subliminal emotional priming. However, building upon our prior research findings (Battaglia et al., 2022a,b), our current study reveals that negative stimuli can enhance action control both when presented as stop signals and when primed before the go signals. Notably, the non-conscious presentation of the emotional prime stimuli ensures that they do not detrimentally capture attention, which would interfere with action control. Contrarily, we demonstrate that the subliminal presentation of negative stimuli enhances task performance in line with several evidences (de Gelder et al., 2005; Yang et al., 2011; Bertini et al., 2013; Zhan and de Gelder, 2019). The effect of the subliminal presentation of a fearful body image on the motor system is not surprising considering that functional magnetic resonance imaging (fMRI) studies have reported non-consciously perceived negative bodies, displayed in the blind field of a cortically blind patient, stimuli managed to elicit extensive cortical activity, encompassing motor and premotor cortices (Van den Stock et al., 2011). We also found that SICI, but not ICF, can predict action control abilities, with participants better in action control demonstrating higher levels of SICI, in line with recent research (He et al., 2019; Chowdhury et al., 2019a,b; Tran et al., 2020; Ding et al., 2021; Loomes et al., 2023). These intriguing findings suggest that the tonic inhibition observed when an individual is not actively engaged in intentional response control (specifically measured at rest before the SST) still serves as a predictor for stopping efficiency in subsequent tasks. Furthermore, they also imply that while action control involves various brain regions (i.e., the Action Inhibition Network; Borgomaneri et al., 2020b), the variability in the SSRT is partially influenced by the variations in local intracortical inhibitory mechanisms within the motor system. This mechanism is believed to be modulated by GABA_A_ neurotransmission (Ziemann et al., 1996). Interestingly, only two previous studies have investigated the potential contribution of glutamatergic projection, mediated by ICF, in action control (Chowdhury et al., 2019a,b; Ding et al., 2021). In line with our results, both investigations found no correlation between the ICF and the SSRT, suggesting that intracortical glutamatergic interneurons may not be involved in the process of response inhibition, but rather during action preparation (Bundt and Huster, 2023). An important limitation of our study is that we did not collect objective measures of awareness. However, the fast presentation of the stimuli (~17 ms), together with the use of the sandwich mask procedures, is generally considered a subliminal presentation (Bar and Biederman, 1998; Harris et al., 2011; Breitmeyer, 2015). Moreover, the subjective measures confirmed that out of 46 participants, only two were aware of the presence of an emotional stimulus. Another issue is the absence of a positive stimulus or another negative control stimulus (e.g., angry body posture) as additional prime stimuli to test possible valence or arousal-related effects. However, previous data (Battaglia et al., 2022a,b) suggest no difference between positive and negative stimuli in influencing the SSRT when used as stop stimuli (but see Mancini et al., 2022 for different results using a go/no-go task). Similarly, we may expect a similar effect using angry bodies as prime stimuli, in line with findings suggesting that high arousal negative emotional states are capable of inhibiting the processing of nontarget information and enhancing selective attention (Finucane, 2011).

Taken together, our data add important information in the framework of the Cognitive vs. Affective Primacy debate (Storbeck and Clore, 2007, for review), supporting the Affective Primacy Hypothesis (Zajonc, 1980, 2000; LeDoux, 1996), based on which emotional information is processed quickly and automatically, before information about ontological kinds. Moreover, our results suggest that SICI can be considered as a potentially useful biomarker for inhibitory control deficit in the clinical setting. Indeed, resting-state intracortical inhibition has been found to be reduced in many disorders with inhibitory control deficits (Greenberg et al., 1998, 2000; Hoegl et al., 2012; Wu et al., 2012). For example, reduced SICI and increased ICF were observed in individuals with attention-deficit/hyperactivity disorder (Hoegl et al., 2012; Wu et al., 2012) and individuals with obsessive-compulsive disorder (Greenberg et al., 1998, 2000). Importantly, most of these deficits are accompanied by problems in emotion perception. Thus, investigations taking into account action control embedding emotional stimuli in clinical populations are highly desirable (Battaglia et al., 2023, 2024; Tanaka et al., 2024).

## Data availability statement

The raw data supporting the conclusions of this article will be made available by the authors, without undue reservation.

## Ethics statement

The studies involving humans were approved by Bioethical Committee of the University of Bologna. The studies were conducted in accordance with the local legislation and institutional requirements. The participants provided their written informed consent to participate in this study.

## Author contributions

TQ: Data curation, Formal analysis, Visualization, Writing – original draft. GI: Investigation, Writing – review & editing. LP: Data curation, Writing – review & editing. PC: Data curation, Formal analysis, Software, Writing – review & editing. SBa: Writing – review & editing. SBo: Conceptualization, Funding acquisition, Investigation, Methodology, Project administration, Resources, Supervision, Writing – original draft, Writing – review & editing.
